# Fetal Adrenal Gland Ultrasound Parameters in Pregnancies with Fetal Growth Restriction Defined by Delphi Criteria: A Prospective Single-Center Case–Control Study

**DOI:** 10.3390/jcm15083082

**Published:** 2026-04-17

**Authors:** Emel Özalp, Özgür Volkan Akbulut, Sadun Sucu, Dilara Sarikaya Kurt, Şevki Çelen, Kadriye Yakut Yücel

**Affiliations:** 1 Department of Obstetrics and Gynecology, Ankara Etlik City Hospital, Ankara 06010, Türkiye; emelozalp@gmail.com; 2 Department of Perinatology, Ankara Etlik City Hospital, Ankara 06010, Türkiye; drssucu@gmail.com (S.S.); sevkicelen@yahoo.com (Ş.Ç.); yakutkadriye@hotmail.com (K.Y.Y.); 3 Department of Obstetrics and Gynecology, Mudanya State Hospital, Bursa 16940, Türkiye; dilarasarikaya30@gmail.com

**Keywords:** fetal growth restriction, fetal adrenal gland, fetal zone, middle adrenal artery, Doppler, ultrasound

## Abstract

**Objective:** This study compared fetal adrenal gland ultrasound parameters between pregnancies complicated by fetal growth restriction (FGR) diagnosed according to Delphi consensus criteria and gestational-age-matched normally grown controls, and interpreted their apparent discriminatory performance cautiously. **Methods:** This prospective single-center case–control study with a cross-sectional ultrasound assessment enrolled 60 singleton pregnancies (30 FGR, 30 controls) between 24 and 41 weeks’ gestation. Controls were recruited contemporaneously from the same unit and had normal fetal biometry and Doppler findings. All examinations were performed using a Voluson E8 system by a single experienced operator; operator blinding to group status was not feasible in routine clinical practice. Standard fetal biometry and Doppler indices (umbilical artery [UA] PI, middle cerebral artery [MCA] PI, uterine artery [UtA] PI) were recorded and the cerebroplacental ratio (CPR) was calculated. Fetal adrenal assessment included the total adrenal gland volume, fetal zone (FZ) width, and middle adrenal artery (MAA) Doppler PI. **Results:** Maternal age, body mass index, and gestational age at scan were similar between groups (*p* > 0.05). Compared with controls, the FGR group had higher UA PI and UtA PI and lower MCA PI and CPR (all *p* < 0.001). Absolute adrenal gland volume was lower in FGR (0.46 ± 0.03 vs. 0.68 ± 0.04 cm^3^; mean difference −0.22 cm^3^, 95% CI −0.24 to −0.20; *p* < 0.001), and FZ width was smaller (median 4.70 vs. 6.55 mm; Hodges–Lehmann shift −1.80 mm, 95% CI −2.00 to −1.70; *p* < 0.001). MAA PI was higher in FGR (2.44 ± 0.14 vs. 1.79 ± 0.12; mean difference 0.65, 95% CI 0.58–0.72; *p* < 0.001). In this selected case–control dataset, adrenal volume, FZ width, and MAA PI each showed apparent complete separation (empirical AUC = 1.00); however, these findings should be interpreted cautiously because absolute adrenal measures were not adjusted for fetal size and such performance may reflect spectrum effects in a relatively small sample. **Conclusions:** In pregnancies with Delphi-defined FGR, absolute fetal adrenal volume and fetal zone width were lower, and MAA PI was higher than in controls. These findings should be considered hypothesis-generating and require external validation in larger multicenter cohorts using standardized and size-adjusted measurement approaches before clinical implementation.

## 1. Introduction

Fetal growth restriction (FGR) is a pathologic condition in which the fetus fails to achieve its biological growth potential and is strongly associated with stillbirth, neonatal morbidity, and long-term cardiometabolic and neurodevelopmental sequelae [1,2,3]. Contemporary definitions and management frameworks emphasize not only fetal size but also Doppler evidence of placental insufficiency, as reflected in the Delphi consensus definition and subsequent international guidelines [4,5].

Umbilical artery (UA) and middle cerebral artery (MCA) Doppler indices and the cerebroplacental ratio (CPR) remain central to fetal surveillance, although their prognostic performance varies by gestational age and clinical context [5,6,7]. This has encouraged interest in complementary sonographic markers that might better characterize fetal adaptation to placental insufficiency.

Beyond cerebral redistribution, chronic hypoxemia and undernutrition in placenta-mediated FGR can induce adaptive circulatory and endocrine changes affecting other organs, including the fetal adrenal glands [8,9,10]. The fetal adrenal gland is a highly vascularized organ with a prominent fetal zone and a key role in the fetoplacental steroidogenic unit and stress response [11,12]. Depending on the timing and severity of placental disease, adrenal adaptation may involve changes in overall size, zonal morphology, and arterial impedance [13,14,15]. 

However, published adrenal studies are heterogeneous. Prior reports have variably examined small-for-gestational-age fetuses, clinically defined IUGR, or FGR; have included different gestational age windows and severity profiles; and have used absolute rather than fetal-size-adjusted adrenal indices [16,17,18,19]. In addition, different studies have focused on different adrenal arteries or measurement planes, which limits comparability and clinical translation. The aim of this study was therefore to evaluate whether fetal adrenal gland ultrasound parameters (adrenal gland volume, fetal zone width, and middle adrenal artery Doppler PI) differ between Delphi-defined FGR and normally grown fetuses in a prospective single-center case–control study with a cross-sectional ultrasound assessment.

## 2. Materials and Methods

Study design and setting: This was a prospective single-center case–control study with a cross-sectional ultrasound assessment performed at the time of recruitment in the Perinatology Unit of a tertiary referral center. The study protocol was approved by the institutional ethics committee (decision date 16 August 2023; decision number AEŞH-EK1-2023-477). Written informed consent was obtained from all participants. Reporting was guided by STROBE recommendations for observational studies [20].

**Participants:** Singleton pregnancies between 24 and 41 weeks’ gestation were eligible. The FGR group consisted of fetuses diagnosed with growth restriction according to the Delphi consensus definition, integrating fetal size (estimated fetal weight and/or abdominal circumference centiles) and Doppler criteria appropriate for gestational age [1,2]. The control group was recruited contemporaneously from the same unit during the same gestational window and included pregnancies with appropriate fetal growth, estimated fetal weight and abdominal circumference between the 10th and 90th centiles, and normal UA, MCA, and uterine artery Doppler findings. Recruitment was consecutive within each group to reduce selection bias.

**Exclusion criteria:** Multiple gestation; maternal systemic disease (e.g., chronic hypertension, diabetes mellitus, thyroid disease, connective tissue disease); chronic medication use; smoking or alcohol use; antenatal infection/chorioamnionitis; fetal structural anomaly or suspected chromosomal abnormality; and major obstetric complications likely to confound fetal hemodynamics (e.g., preeclampsia) were excluded.

**Ultrasound protocol and operator blinding:** All examinations were performed by a single experienced obstetric sonographer (S.S., 10 years of experience) using a GE Voluson E8 ultrasound system equipped with a C1-5-RS convex transducer (GE Healthcare Austria GmbH & Co OG, Zipf, Austria). Standard fetal biometry included abdominal circumference (AC). Doppler velocimetry was obtained from the UA, MCA, and uterine artery (UtA), and pulsatility index (PI) values were recorded. CPR was calculated as MCA PI/UA PI. Biometrics and Doppler measurements were conducted in accordance with the Delphi consensus criteria and the International Society of Ultrasound in Obstetrics and Gynecology (ISUOG) Practice Guidelines. Because group assignment depended on fetal biometry and Doppler findings obtained in the clinical examination, operator blinding to case/control status was not feasible; this is acknowledged as a limitation.

**Figure 1:**
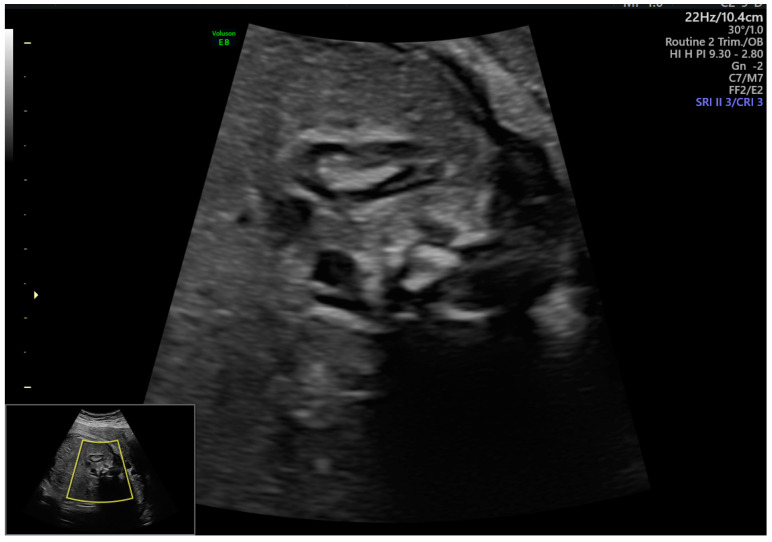
Sonographic assessment of fetal adrenal gland morphology.

Ultrasound Assessment of the Fetal Adrenal Gland: The fetal adrenal gland was identified as a crescent-shaped structure located superior to the fetal kidney (Figure 1). Measurements were obtained in the sagittal or mildly oblique transverse plane, ensuring clear delineation of the adrenal gland borders. Care was taken to avoid bone-related acoustic shadowing and oblique sections that could interfere with accurate assessment. Measurement approaches were consistent with methods described in prior fetal adrenal ultrasound studies [12,16,18].

Representative two-dimensional ultrasound imagery showing sagittal visualization of the fetal adrenal gland above the fetal kidney. The maximum longitudinal diameter of the adrenal gland is measured at the level where glandular borders are most clearly delineated. Care was taken to avoid oblique imaging planes and acoustic artifacts during measurement.

The maximum longitudinal length, transverse width, and anteroposterior thickness of the adrenal gland were measured. Total adrenal gland volume was calculated using the ellipsoid formula (length × width × thickness × 0.523). Each measurement was obtained at least three times, and the mean value was used for statistical analysis. All measurements were performed in the absence of fetal breathing or gross fetal movements to minimize motion-related variability. Each participant contributed one cross-sectional study scan only. Although repeated measurements were averaged, formal intraobserver and interobserver reproducibility analyses were not performed, because all examinations were acquired by a single operator within routine clinical workflow.

**Fetal Zone Measurement:** In the transverse plane of the adrenal gland, the centrally located hypoechoic region was identified as the fetal zone, while the peripheral hyperechoic rim was defined as the definitive cortex. The maximum diameter of the fetal zone and the total adrenal gland diameter were measured. The fetal zone ratio was calculated as the ratio of the fetal zone diameter to the total adrenal gland diameter. Because archived raw data were insufficient for robust size-normalized recalculation and because the fetal zone ratio showed less clinically relevant separation than the fetal zone width, the present analysis emphasizes the fetal zone width as the principal zonal parameter.

**Figure 2:**
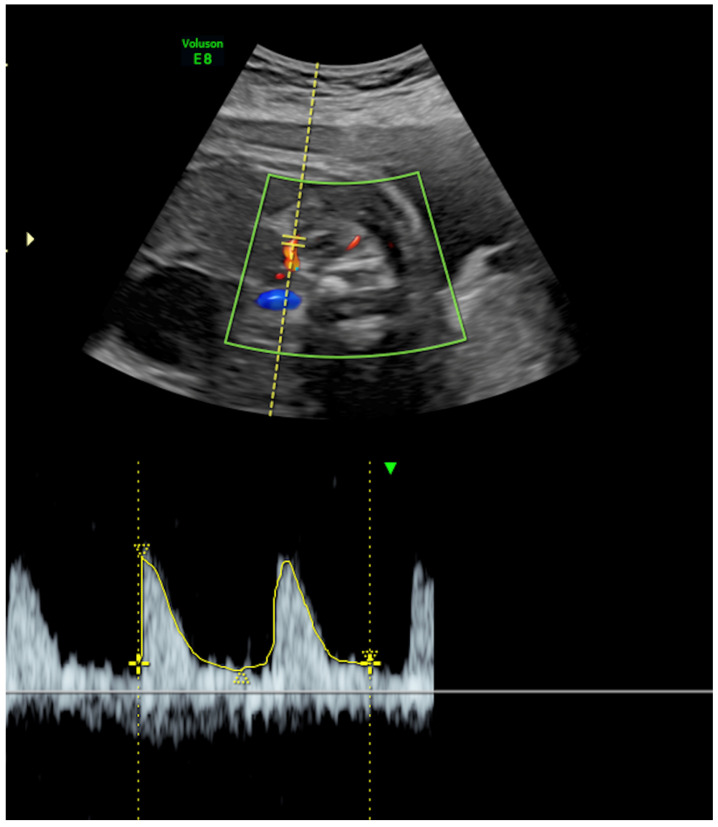
Middle adrenal artery Doppler velocimetry.

**Adrenal Artery Doppler Assessment:** The middle adrenal artery (MAA) was identified using color Doppler ultrasonography. Doppler settings were optimized by adjusting the pulse repetition frequency (PRF) between 0.5 and 1.0 kHz and maintaining a low wall motion filter. Doppler measurements were obtained with an insonation angle of less than 30 degrees relative to the direction of blood flow. Pulsatility index (PI) and resistance index (RI) values were recorded from at least three consecutive uniform waveforms, and the average values were used for analysis (Figure 2). In the final manuscript, PI is emphasized because it showed the most consistent between-group signal, whereas RI was evaluated exploratorily and is not presented as a principal finding.

In representative color and pulsed-wave Doppler ultrasound imagery showing middle adrenal artery (MAA) velocimetry, the adrenal gland is identified above the fetal kidney, and the MAA is visualized within the gland using color Doppler. Spectral Doppler measurements were obtained with an insonation angle < 30°, low wall motion filter, and optimized pulse repetition frequency for low-resistance flow. The pulsatility index (PI) was derived from at least three consecutive cardiac cycles with uniform waveforms.

**Outcomes:** The primary outcomes were between-group differences in adrenal gland volume, fetal zone width, and MAA PI. Secondary outcomes included the delivery week, birthweight, Apgar scores, antenatal corticosteroid exposure (ACS), and neonatal intensive care unit (NICU) admission. The fetal zone ratio and MAA RI were assessed as exploratory variables but were not emphasized among the primary findings because they did not materially strengthen discrimination and were considered more susceptible to measurement variability.

**Sample size:** An a priori calculation was performed in G*Power, 3.1.9.7 [21]. Based on the large standardized differences reported in earlier pilot adrenal ultrasound studies [14,16], we assumed an effect size of approximately Cohen’s d = 1.0, two-sided alpha = 0.05, and power = 0.95, which corresponded to a minimum of 27 pregnancies per group. We therefore enrolled 30 pregnancies per group to improve precision and allow for potential attrition or non-evaluable measurements.

**Statistical analysis:** Continuous variables were assessed for normality (Shapiro–Wilk) and summarized as mean ± SD or median [IQR]. Between-group comparisons used Welch’s *t*-test for approximately normally distributed variables or the Mann–Whitney U test otherwise. Categorical variables were compared using Fisher’s exact test. ROC curves were generated for the three principal adrenal parameters to describe apparent discrimination of FGR within the present study sample. Optimal thresholds were estimated using Youden’s index. Because the observed thresholds classified 30/30 cases or controls correctly, exact binomial 95% confidence intervals were calculated for sensitivity and specificity. No multivariable modeling was attempted because of the modest sample size and complete separation of the main adrenal parameters. A two-sided *p* value < 0.05 was considered statistically significant. Analyses were performed in Python 3.x.y.

## 3. Results

Participant characteristics and ultrasound findings are summarized in Table 1. Maternal age, BMI, and gestational age at the time of scan were similar between groups (all *p* > 0.05), suggesting that major maternal anthropometric differences did not account for the observed adrenal findings. As expected for placenta-mediated FGR, the FGR group demonstrated higher UA PI and UtA PI and lower MCA PI and CPR compared with controls (all *p* < 0.001).

Fetal adrenal parameters differed markedly between groups. The absolute adrenal gland volume was lower in FGR (0.46 ± 0.03 vs. 0.68 ± 0.04 cm^3^; mean difference −0.22 cm^3^, 95% CI −0.24 to −0.20; *p* < 0.001). The FZ width was smaller in FGR (median 4.70 [4.53–4.90] vs. 6.55 [6.22–6.80] mm; Hodges–Lehmann shift −1.80 mm, 95% CI −2.00 to −1.70; *p* < 0.001). MAA PI was higher in FGR (2.44 ± 0.14 vs. 1.79 ± 0.12; mean difference 0.65, 95% CI 0.58–0.72; *p* < 0.001). Exploratory analyses of the fetal zone ratio and MAA RI did not provide findings as robust as those of the fetal zone width and MAA PI and therefore are not emphasized among the principal results.

Perinatal outcomes are shown in Table 2. The FGR group delivered earlier and had a lower birthweight and lower 1 and 5 min Apgar scores (all *p* < 0.001). NICU admission occurred in 53.3% of the FGR group and in 0% of controls (*p* < 0.001). Antenatal corticosteroid exposure was more common in FGR (46.7% vs. 0%; *p* < 0.001). These findings are clinically coherent with the greater severity of placental insufficiency in the FGR group.

**Figure 3:**
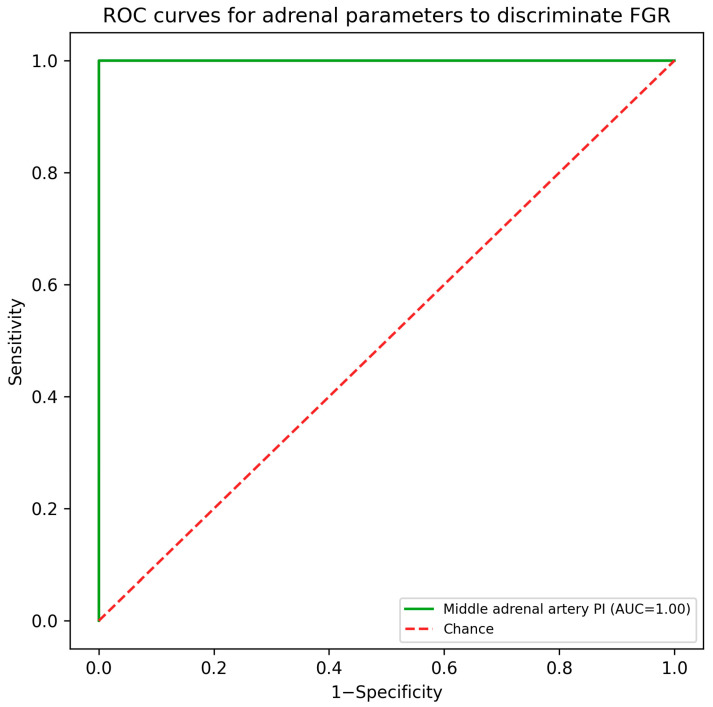
ROC curves for adrenal parameters to discriminate FGR in the study sample. Because all three parameters showed complete separation in this dataset, the curves converge near the upper-left corner. The figure illustrates apparent within-sample discrimination rather than validated generalizable predictive performance.

Diagnostic performance: In this case–control dataset, adrenal volume, FZ width, and MAA PI each showed empirical complete separation between groups (AUC = 1.00). Example cut-offs that achieved 100% sensitivity and specificity in the study sample were adrenal volume ≤ 0.56 cm^3^, FZ width ≤ 5.55 mm, and MAA PI ≥ 2.09 (Table 3; Figure 3). Because each threshold classified 30/30 affected fetuses and 30/30 controls correctly, the exact 95% confidence interval for both sensitivity and specificity was 88.4–100.0%. These estimates represent apparent performance in the present dataset and should not be interpreted as validated screening accuracy.

## 4. Discussion

In this prospective single-center case–control study with a cross-sectional ultrasound assessment, fetuses with Delphi-defined FGR demonstrated substantial differences in fetal adrenal ultrasound parameters compared with normally grown controls. The absolute adrenal gland volume and fetal zone width were lower, whereas MAA PI was higher in the FGR group. A similar maternal age, BMI, and gestational age at examination reduce the likelihood that the findings were driven by clear baseline imbalance, while the accompanying Doppler profile (higher UA and uterine artery PI, lower MCA PI and CPR) and adverse perinatal outcomes support the internal consistency of the FGR phenotype in our cohort.

Placenta-mediated FGR is driven largely by impaired uteroplacental perfusion, chronic fetal hypoxemia, and maladaptive placental development [6]. Fetal cardiovascular adaptation classically includes increased placental resistance (higher UA PI) and cerebral vasodilation (lower MCA PI), resulting in a reduced CPR [2,3,4]. The adrenal glands are also plausible participants in this adaptive response because the fetal adrenal gland is both highly vascular and endocrinologically active, with a large fetal zone that contributes to steroid precursor production in the fetoplacental unit [7,8,9]. Chronic hypoxemia may therefore influence adrenal perfusion and zonal maturation in addition to the overall gland size.

Earlier Doppler studies of adrenal arteries in small-for-gestational-age or growth-restricted fetuses suggested reduced impedance as part of an adrenal-sparing effect [10,11]. More recent work has explored zone-specific adrenal perfusion and sonographic adrenal biometry in FGR [12,16,18,19]. However, the literature remains heterogeneous with respect to case definition, gestational age at assessment, severity of placental disease, and the use of absolute versus size-adjusted adrenal measures.

Several studies reported an enlarged adrenal size or higher adrenal-to-abdominal ratios in FGR/IUGR, findings interpreted as relative adrenal sparing [17,18,19]. Heese et al. observed an increased adrenal cortex width and adrenal gland ratio in FGR [17], Pattamathamakul et al. reported larger weight-corrected adrenal volumes in FGR [18], and Martinelli et al. demonstrated a higher adrenal/abdominal circumference ratio together with biochemical evidence of stress activation [19]. In contrast, we observed a smaller absolute adrenal volume and fetal zone width. The most likely explanation is methodological rather than biologically contradictory: our adrenal parameters were not normalized to fetal size. Because fetuses with FGR are smaller overall, absolute adrenal dimensions may primarily reflect global fetal biometry rather than selective adrenal adaptation. This is a major limitation of the present study and should be a priority for future work.

We found higher MAA PI in FGR, indicating higher vascular resistance in the vessel supplying the fetal zone. Prior studies have reported heterogeneous MAA Doppler findings in FGR [16,18]. Uyan Hendem et al. reported lower MAA PI values in FGR compared with controls [16], whereas our findings suggest the opposite direction. Discrepancies may be related to differences in case definition, the gestational age and severity spectrum of included fetuses, the arterial segment sampled, Doppler acquisition settings, and whether results are expressed as raw indices, ratios, or gestational-age-adjusted values. These considerations underline the need for standardized acquisition protocols and gestational-age-specific reference frameworks.

Although the absence of overlap in adrenal parameters suggests possible diagnostic value, complete separation in a relatively small single-center case–control sample must be interpreted very cautiously. Case–control studies compare clearly affected pregnancies with clearly normal controls and therefore are particularly prone to spectrum effects and optimistic estimates of discrimination. Consecutive recruitment may have reduced but did not eliminate selection bias. Accordingly, the observed empirical AUC of 1.00 should be viewed as an apparent within-sample finding rather than proof of clinical screening performance or external validity.

From a clinical perspective, fetal adrenal measurements should currently be regarded as exploratory adjuncts rather than replacements for established FGR surveillance based on fetal biometry, UA, MCA, and CPR. Before any routine implementation, it will be necessary to show that adrenal biometry and adrenal artery Doppler add incremental information beyond standard Doppler parameters, are reproducible across operators and platforms, and remain informative after adjustment for fetal size.

Future research should adopt longitudinal designs in women at risk of FGR across different gestational ages so that dynamic changes in adrenal morphology and MAA Doppler can be tracked over time and related to delivery timing, neonatal condition, NICU admission, and longer-term outcomes. Multicenter studies are also needed to develop and validate standardized protocols for adrenal gland plane selection, zone delineation, Doppler sampling site, repeatability testing, and gestational-age- or size-adjusted indices.

Strengths of this study include prospective recruitment, use of Delphi criteria, and performance of all scans on a uniform ultrasound platform by an experienced operator. Important limitations include the modest sample size, single-center design, case–control structure, lack of formal operator blinding, lack of intraobserver and interobserver reproducibility assessment, absence of fetal-size-adjusted adrenal measurements, and lack of serial longitudinal measurements. These limitations restrict generalizability and support a conservative interpretation of the findings.

## 5. Conclusions

Compared with normally grown fetuses, Delphi-defined FGR was associated with a lower absolute fetal adrenal volume and fetal zone width and higher MAA PI. However, because the measurements were absolute, obtained in a small single-center case–control sample, and not subjected to formal reproducibility testing, these results should be considered preliminary. For clinicians, adrenal assessment may serve as an exploratory adjunct when evaluating pregnancies with suspected placental insufficiency, but it should not replace standard surveillance based on fetal biometry and established Doppler indices. Larger multicenter longitudinal studies using standardized and size-adjusted measurement approaches are required before these parameters can be recommended for routine clinical practice.

## Figures and Tables

**Table 1:** Maternal characteristics, fetal biometry, Doppler findings, and adrenal ultrasound parameters at examination.

Variable	Control (*n* = 30)	FGR (*n* = 30)	*p*
Maternal age, years	34.50 [27.50–39.50]	30.50 [24.25–38.75]	0.363
BMI, kg/m^2^	30.00 [29.00–31.00]	30.00 [29.00–31.00]	0.891
Gestational age at scan, weeks	32.46 ± 2.04	32.71 ± 1.83	0.619
Abdominal circumference, mm	283.50 [273.50–291.50]	242.00 [227.50–246.00]	<0.001
Umbilical artery PI	1.03 [0.96–1.11]	1.90 [1.79–2.02]	<0.001
Middle cerebral artery PI	1.79 [1.68–1.86]	1.15 [1.05–1.22]	<0.001
CPR	1.72 ± 0.18	0.60 ± 0.07	<0.001
Uterine artery PI	0.65 [0.60–0.70]	0.90 [0.80–1.00]	<0.001
Adrenal gland volume, cm^3^	0.68 ± 0.04	0.46 ± 0.03	<0.001
Fetal zone width, mm	6.55 [6.22–6.80]	4.70 [4.53–4.90]	<0.001
Middle adrenal artery PI	1.79 ± 0.12	2.44 ± 0.14	<0.001

Note. Values are presented as mean ± SD or median [IQR], according to distribution. *p* values were obtained using Welch’s *t*-test for approximately normally distributed variables and the Mann–Whitney U test for non-normally distributed variables. BMI, body mass index; CPR, cerebroplacental ratio; FGR, fetal growth restriction; PI, pulsatility index.

**Table 2:** Delivery and neonatal outcomes.

Variable	Control (*n* = 30)	FGR (*n* = 30)	*p*
Gestational age at delivery, weeks	38.50 [38.00–40.00]	36.50 [36.00–37.00]	<0.001
Birthweight, g	3000.00 [2758.50–3164.50]	1758.00 [1617.50–1833.75]	<0.001
Apgar score at 1 min	7.50 [7.00–8.00]	5.00 [4.00–6.00]	<0.001
Apgar score at 5 min	9.00 [9.00–9.00]	7.00 [6.25–8.00]	<0.001
NICU admission, *n* (%)	0/30 (0.0%)	16/30 (53.3%)	<0.001
Antenatal corticosteroid exposure, *n* (%)	0/30 (0.0%)	14/30 (46.7%)	<0.001

Note. Values are presented as median [IQR] or *n* (%). *p* values were obtained using the Mann–Whitney U test for continuous variables and Fisher’s exact test for categorical variables. ACS, antenatal corticosteroid exposure; FGR, fetal growth restriction; NICU, neonatal intensive care unit.

**Table 3:** ROC-derived thresholds for fetal adrenal parameters to discriminate FGR from controls.

Parameter	Threshold Indicating FGR	AUC	Sensitivity	Specificity
Adrenal volume	≤0.56 cm^3^	1.00	1.00	1.00
Fetal zone width	≤5.55 mm	1.00	1.00	1.00
MAA PI	≥2.09	1.00	1.00	1.00

Note. Thresholds were selected using Youden’s index from receiver operating characteristic analysis. In this case–control sample, the empirical AUC was 1.00 for each parameter because of complete separation. At the listed thresholds, sensitivity and specificity were 100%; exact 95% CIs for both estimates were 88.4–100.0%, based on 30/30 correctly classified cases or controls. Performance estimates were derived from the present case–control dataset and require external validation. AUC, area under the receiver operating characteristic curve; FGR, fetal growth restriction; MAA PI, middle adrenal artery pulsatility index.

## Data Availability

Due to hospital policies, patient data and study materials cannot be shared. However, the data are available from the corresponding author upon reasonable request.
